# Thulium fibre laser endoscopic enucleation of the prostate (THUFLEP) learning curve assessment: A retrospective analysis of the experience of a single surgeon

**DOI:** 10.1002/bco2.70116

**Published:** 2025-12-03

**Authors:** Sulaiman Goudaimy, Louis Lenfant, Christophe Vaessen, Jérôme Parra, Emmanuel Chartier‐Kastler, Aurélien Beaugerie, Pierre Mozer, Quentin Dubourg, Thomas Seisen, Morgan Roupret, Ugo Pinar

**Affiliations:** ^1^ Sorbonne University, GRC 5, Predictive Onco‐Urology, APHP, Pitié‐Salpêtrière Hospital, Department of Urology Paris France

**Keywords:** benign prostatic hyperplasia, lower urinary tract symptoms, mini‐invasive surgery, prostate enucleation

## Abstract

**Objectives:**

To assess the learning curve of thulium fibre laser enucleation of the prostate (THUFLEP).

**Patients and methods:**

We conducted a retrospective study including the first 111 patients treated with THUFLEP by a single surgeon. The operator had no prior experience in prostate enucleation and performed the first two procedures under the supervision of an expert. Initial 68 cases were performed using the three‐lobe technique, and the later cases were done as en bloc enucleation with early apex release (*n* = 43). The learning curve was analysed in terms of temporal changes in the following variables: enucleation time, morcellation time, occurrence of intraoperative complications (IOCs) and achievement of pentafecta (enucleation + morcellation <90 min, a complete enucleation without complications and stress urinary incontinence at 3 months) in four consecutive patients.

**Results:**

A total of 111 consecutive patients were included, with a mean age of 71.5 years (SD = 10.8), a mean total PSA of 7.9 ng/ml (SD = 8.4) and a mean prostate volume of 99.1 cc (SD = 35.9). The mean enucleation and morcellation times were 65.9 min (SD = 36.2) and 15.5 min (SD = 13.5), respectively. There were 13 postoperative complications (11.7%), and only one required reoperation for clot evacuation. The pentafecta was achieved in 66 patients (59.5%) and was reached in four consecutive patients after the 38th case. Operative time reached a plateau after approximately 60 procedures. The average enucleation efficiency was 1.1 g/min (SD = 0.7), and the plateau had not yet been reached after 111 patients.

**Conclusions:**

In our experience, we observed a learning curve of 40–60 cases for THUFLEP. This plateau was reached after a relatively long learning curve; a more structured and supervised training programme may help improve these learning curves. Lastly, the complication rate was low from the beginning of the surgical experience, and the pentafecta was achieved in more than half of the patients.

## 1 INTRODUCTION

Benign prostatic hyperplasia (BPH), or prostate enlargement, is a common condition affecting a substantial portion of the ageing male population, often leading to lower urinary tract symptoms (LUTS) that can significantly impact a patient's quality of life. In the United States, studies have shown that BPH affects more than 80% of men over the age of 70.^1^

Endoscopic enucleation of the prostate (EEP) is currently the recommended treatment for patients with BPH‐induced LUTS presenting surgical indications.^2^, ^3^ EEP efficiency and safety have been demonstrated compared to more conventional procedures.^4^, ^5^ Consequently, various methods have been described and different laser energies can be used such as holmium laser, thulium‐Yag laser or thulium fibre laser (TFL).^6^ Although the surgical technique remains surgeon dependent, the choice of laser in EEP could have an impact on the perioperative outcomes.^7^

Another laser choice criterion should be the learning curve. Indeed, the EEP learning curve is steep and hard to reach, which may discourage urologists new to the technique. The holmium EEP (HOLEP) learning curve has been widely studied, and it is estimated that 50 cases are needed to reach a plateau.^8^, ^9^ However, scarce data are available regarding the thulium fibre laser enucleation of the prostate (THUFLEP) learning curve. The TFL with a shorter wavelength (1940 nm) at the peak of the water absorption spectrum could allow better energy absorption in tissues and better haemostasis.^10^ Knowing those laser energy properties, we could presume that the learning curve plateau could be easier to reach.

This study aims to evaluate the THUFLEP learning curve of a single surgeon in a high‐volume academic centre.

## 2 METHODS

### 2.1 Population and data collection

All consecutive patients who underwent THUFLEP surgery by the same surgeon were retrospectively included in this study. Included patients had a surgical indication according to available guidelines. Patients were included during 9 months. Demographic, preoperative, perioperative and functional outcome data were collected prospectively. The size of the prostate was measured either with ultrasonography or with MRI. Patients without a preoperative catheter also underwent uroflowmetry and post‐void residual estimation using ultrasonography. The severity of BPH‐related symptoms was assessed using the IPSS questionnaire. Patients in acute urinary retention were either catheterized or received intermittent self‐catheterization until surgery. After THUFLEP, catheters were removed on day 1 if the urine was not too red, and patients were discharged the same day if they had spontaneous micturition. Patients had a follow‐up 3 months after surgery.

### 2.2 Surgical technique

THUFLEP was performed by one urologist who had never performed EEP but who had 5 years of experience in trans‐urethral resection of the prostate. The first two THUFLEP procedures were performed under the supervision of an expert. We used a TFL drive laser generator (Coloplast©) with a 550‐μm fibre using the following settings (pulse mode, 1.5 J, 40 Hz for enucleation and 1 J, 20 Hz for coagulation). We used a Storz laser resectoscope (Ch26) and a Storz morcellator. The surgeon began his experience with the three‐lobe enucleation technique and, when he felt confident, began en bloc enucleation with early apical release.

### 2.3 Outcomes

The learning curve was assessed using two different methods. Firstly, according to the previously published pentafecta,^11^ pentafecta was defined as the combination of enucleation time and morcellation time <90 min, the absence of intraoperative conversion to TURP or open surgery, the absence of any 30‐day complications and the absence of stress urinary incontinence at 3 months. The learning curve was overcome when four consecutive patients achieved pentafecta. Secondly, we also considered that the learning curve was overcome when a plateau was reached for operative time, enucleation time and efficiency. As a secondary outcome, we tried to look for predictors of pentafecta outcome.

### 2.4 Statistical analysis

Statistical analysis was performed using R version 3.6.2. (2009–2019 RStudio, Inc.). Quantitative variables are described as mean and standard deviation (SD) and qualitative variables as numbers and percentages. The cohort was then divided into two groups according to the number of patients needed to achieve four consecutive pentafecta and perioperative outcomes were compared. To compare categorical variables, Pearson's Chi2 test was used, and to compare continuous variables the Wilcoxon rank‐sum test. As the data did not follow a normal distribution, we opted for the Mann–Whitney *U* test to compare continuous variables. Learning curves were evaluated by assessing operative time, enucleation time and efficiency for consecutive patients. To evidence pentafecta predictors we used univariate and multivariate logistic regression. Significant results were set for a *p* value <0.05.

## 3 RESULTS

### 3.1 Patient's characteristics

Overall, 111 consecutive patients were included in this study (Table 1). The mean (SD) age was 71.5 years (SD = 10.8), the mean (SD) total PSA was 7.9 ng/ml (SD = 8.4), and the mean prostate volume was 99.1 cc (SD = 35.9). Of them, nine (8.1%) had therapeutic anticoagulation, and 27 (24.3%) had an antiplatelet agent.

**TABLE 1:** Demographic and preoperative data.

Variable	Study cohort (*N* = 111)
Demographic data	
Age in years, mean (SD)	71.5 (10.8)
BMI in kilo/m^2^, mean (SD)	26.8 (4.1)
ASA score, *n* (%)	
1	27 (24.3)
2	66 (59.5)
3	18 (16.2)
4	(0.0)
5	(0.0)
Therapeutic anticoagulation, *n* (%)	9 (8.1)
Antiplatelet drug, *n* (%)	27 (24.3)
Diabetes, *n* (%)	20 (18.0)
History of BPH surgical treatment, *n* (%)	10 (9.0)
History of prostate cancer, *n* (%)	15 (13.5)
Preoperative data	
Surgical motive, *n* (%)	
Urinary retention	43 (38.7)
Dysuria	28 (25.2)
Increased daytime frequency	22 (19.8)
Urgency	5 (4.5)
Other	13 (11.7)
Prostate medications, *n* (%)	
Alpha‐blockers (alone or combined)	99 (89.2)
5 ARIs (alone or combined)	12 (10.8)
Preoperative voiding, *n* (%)	
Spontaneous	72 (64.9)
Urethral catheter	26 (23.4)
Self‐catheterization	13 (11.7)
PSA in ng/dl, mean (SD)	7.9 (8.4)
Prostate volume in cc, mean (SD)	99.1 (35.9)
IPSS, mean (SD)	17.0 (7.6)
IPSS QoL, mean (SD)	4.0 (1.6)
Maximum urinary flow in ml/S, mean (SD)	9.2 (5.2)
Post‐void residual urine in ml, mean (SD)	204.2 (241.1)

### 3.2 Perioperative outcomes

In most procedures, the surgeon used the trilobe enucleation technique (*n* = 68, 61.3%). In the cohort, the mean (SD) enucleation and morcellation times were 65.9 min (36.2) and 15.5 min (13.5), respectively (Table 2). There were no major intraoperative incidents reported; one procedure was stopped during morcellation due to a morcellator breakdown, and a sub‐trigonal perforation was observed in a second procedure. There were two (1.8%) conversions in the cohort: one conversion to TURP for uncontrolled bleeding and one transvesical approach due to a massive adenoma (250 cc). The mean (SD) total enucleated prostate weight was 58.1 g (32.9).

**TABLE 2:** Perioperative data.

Variable	Study cohort (*N* = 111)
Perioperative data	
Enucleation technique, *n* (%)	
En bloc	43 (38.7)
Trilobe	68 (61.3)
Concomitant stone treatment, *n* (%)	6 (5.4)
Surgical durations in minutes, mean (SD)	
Enucleation duration	65.9 (36.2)
Morcellation duration	15.5 (13.5)
Laser duration	29.7 (13.3)
Overall duration	98.0 (48.0)
Energy used in KJ, mean (SD)	97.5 (40.6)
EAUiaic score, *n* ^12^ (%)	
0	108 (97.3)
1	2 (1.8)
2	1 (0.9)
3	0 (0.0)
4	0 (0.0)
5	0 (0.0)
Enucleated prostate weight in grams, mean (SD)	58.1 (32.9)
Conversion, *n* (%)	2 (1.8)
Postoperative data	
Length of hospitalization in days, mean (SD)	1.8 (1.8)
Time to catheter removal in days, mean (SD)	1.4 (1.3)
Clavien–Dindo during hospitalization, *n* (%)	
1	2 (1.8)
2	9 (8.1)
3	1 (0.9)
4	1 (0.9)
5	0 (0.0)
Catheter removal failure, *n* (%)	
During hospitalization	2 (1.8)
At 3 months	0 (0.0)
Pathology results, *n* (%)	
BPH	102 (91.9)
Prostate cancer	8 (7.2)
3‐month unplanned readmission, *n* (%)	1 (0.9)

Regarding postoperative outcomes, the mean (SD) length of stay was 1.8 days (1.8), and there were 13 (11.7%) immediate postoperative complications, most of which were graded Clavien 2 (*n* = 9, 8.1%). At 3 months, seven patients (6.3%) reported stress urinary incontinence (Table 3).

**TABLE 3:** Postoperative functional results.

Variable	Study cohort (*N* = 111)
IPSS, mean (SD)	8.6 (5.8)
IPSS QoL, mean (SD)	1.8 (1.6)
Stress urinary incontinence, *n* (%)	7 (6.3)
Urgency, *n* (%)	25 (22.5)
Qmax in ml/s, mean (SD)	21.3 (11.9)
Pentafecta, *n* (%)	66 (59.5)
PSA in ng/ml, mean (SD)	1.3 (1.6)

### 3.3 Learning curve

The pentafecta was achieved in 66 patients (59.5%) and was reached in four consecutive patients after the 38th case. Univariate logistic regression analysis evidenced that a high preoperative prostate volume and high patient's body mass index (BMI) were predictors of pentafecta failure. Multivariate analysis confirmed that BMI and preoperative prostate volume were two predictors of pentafecta failure (respective OR [IQR] = 0.86 [0.76–0.95], *p* = 0.005 and OR [IQR] = 0.98 [0.95–0.99], *p* = 0.008).

According to the learning curve, the first 40 patients were compared with the subsequent patients. Demographic data were well balanced, especially for BMI and preoperative prostate volume (Table 4). Operative, enucleation and morcellation times were significantly lower in the group of patients who had surgery after reaching the learning curve. There were no differences regarding length of stay, time to catheter removal or postoperative complications.

**TABLE 4:** Comparison of perioperative outcomes between the first 40 patients and the rest of the cohort.

Variable	Group 1 (40 first patients)	Group 2 (*N* = 71)	*p* value
Demographic data			
Age in years, mean (SD)	68.8 (13.5)	72.9 (8.6)	0.06
BMI in kilo/m^2^, mean (SD)	27.4 (4.4)	26.5 (3.9)	0.3
ASA score, *n* (%)			0.9
1	10 (25)	17 (23.9)	
2	23 (57.5)	43 (60.6)	
3	7 (17.5)	11 (15.5)	
4	0 (0.0)	0 (0.0)	
5	0 (0.0)	0 (0.0)	
Therapeutic anticoagulation, *n* (%)	2 (5.0)	7 (9.9)	0.6
Antiplatelet drug, *n* (%)	6 (15.0)	21 (29.6)	0.1
Diabetes, *n* (%)	10 (25)	10 (14.1)	0.2
Preoperative data			
Preoperative voiding, *n* (%)			0.7
Spontaneous	25 (62.5)	47 (66.2)	
Urethral catheter	10 (25.0)	16 (22.5)	
Self‐catheterization	5 (12.5)	8 (11.3)	
PSA in ng/dl, mean (SD)	8.6 (10.0)	7.6 (7.5)	0.6
Prostate volume in cc, mean (SD)	94.5 (29.8)	101.7 (38.9)	0.3
Perioperative data			
Enucleation technique, *n* (%)			<0.01
En bloc	2 (5.0)	41 (57.7)	
Trilobe	38 (95)	30 (42.3)	
Surgical durations in minutes, mean (SD)			
Enucleation duration	93.5 (42.6)	50.4 (19.2)	<0.01
Morcellation duration	20.9 (17.5)	12.4 (9.5)	<0.01
Laser duration	39.7 (15.5)	24.6 (8.4)	<0.01
Overall duration	131.9 (56.8)	78.9 (28.4)	<0.01
Energy used in KJ, mean (SD)	126.4 (46.1)	82.6 (27.6)	<0.01
Enucleated prostate weight in grams, mean (SD)	53.1 (27.2)	62.1 (37.7)	0.2
Conversion, *n* (%)	1 (2.5)	1 (1.4)	1.0
Postoperative outcomes			
Length of hospitalization in days, mean (SD)	1.9 (2.7)	1.7 (0.9)	0.5
Time to catheter removal in days, mean (SD)	1.6 (1.7)	1.7 (1.9)	0.6
Clavien–Dindo during hospitalization, n (%)			0.3
1	0 (0.0)	2 (2.8)	
2	3 (7.5)	6 (8.5)	
3	1 (2.5)	0 (0.0)	
4	1 (2.5)	0 (0.0)	
5	0 (0.0)	0 (0.0)	

**FIGURE 1:**
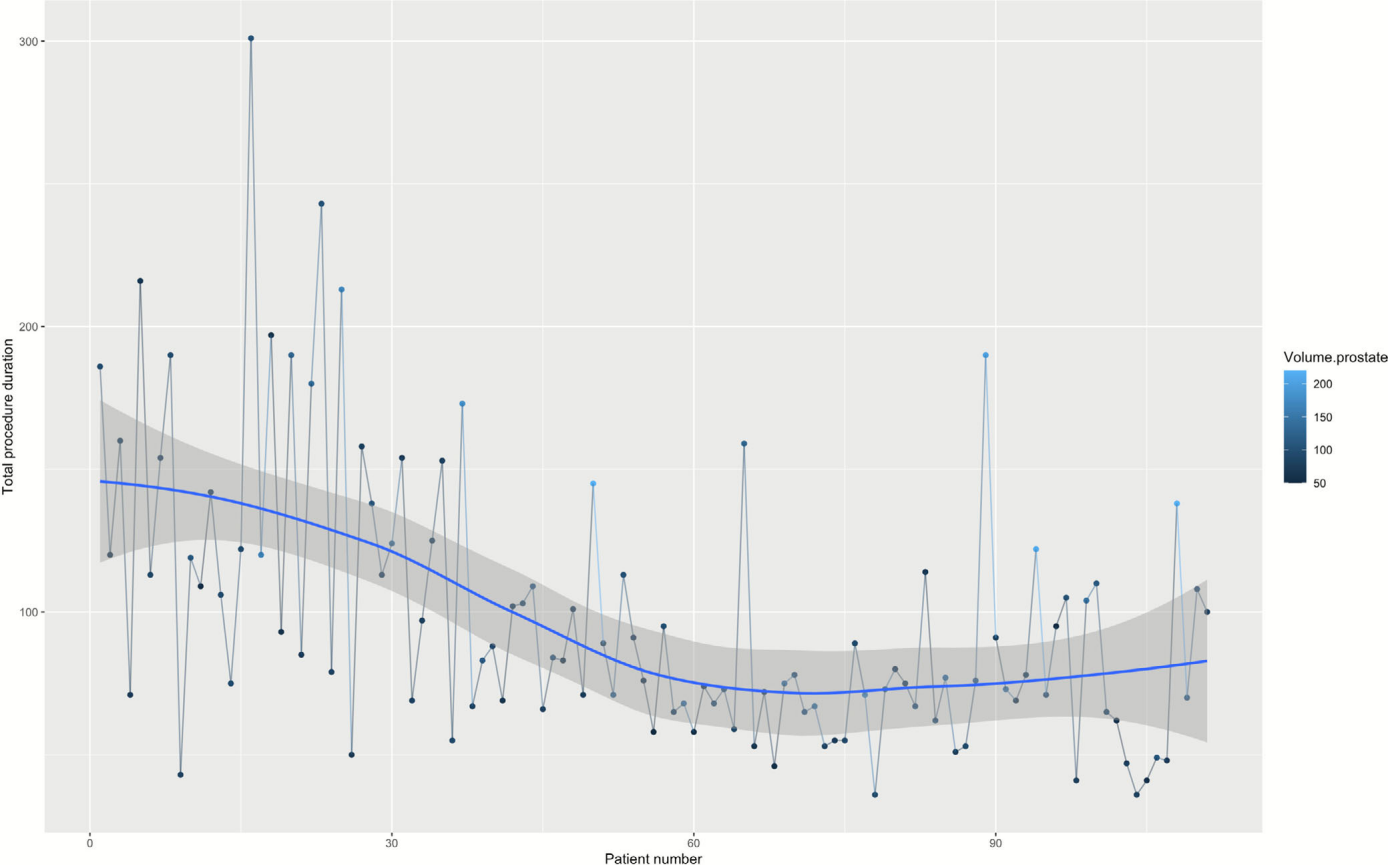
Evolution of total operative time over consecutive patients' operative time decreased during the first procedures to reach a plateau after 60 procedures.

**FIGURE 2:**
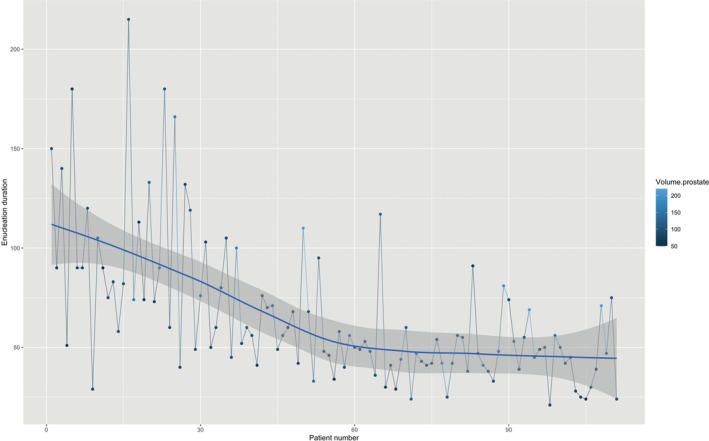
Evolution of enucleation time over consecutive patients. Enucleation time decreased during the first procedures to reach a plateau after approximatively 60 procedures.

**FIGURE 3:**
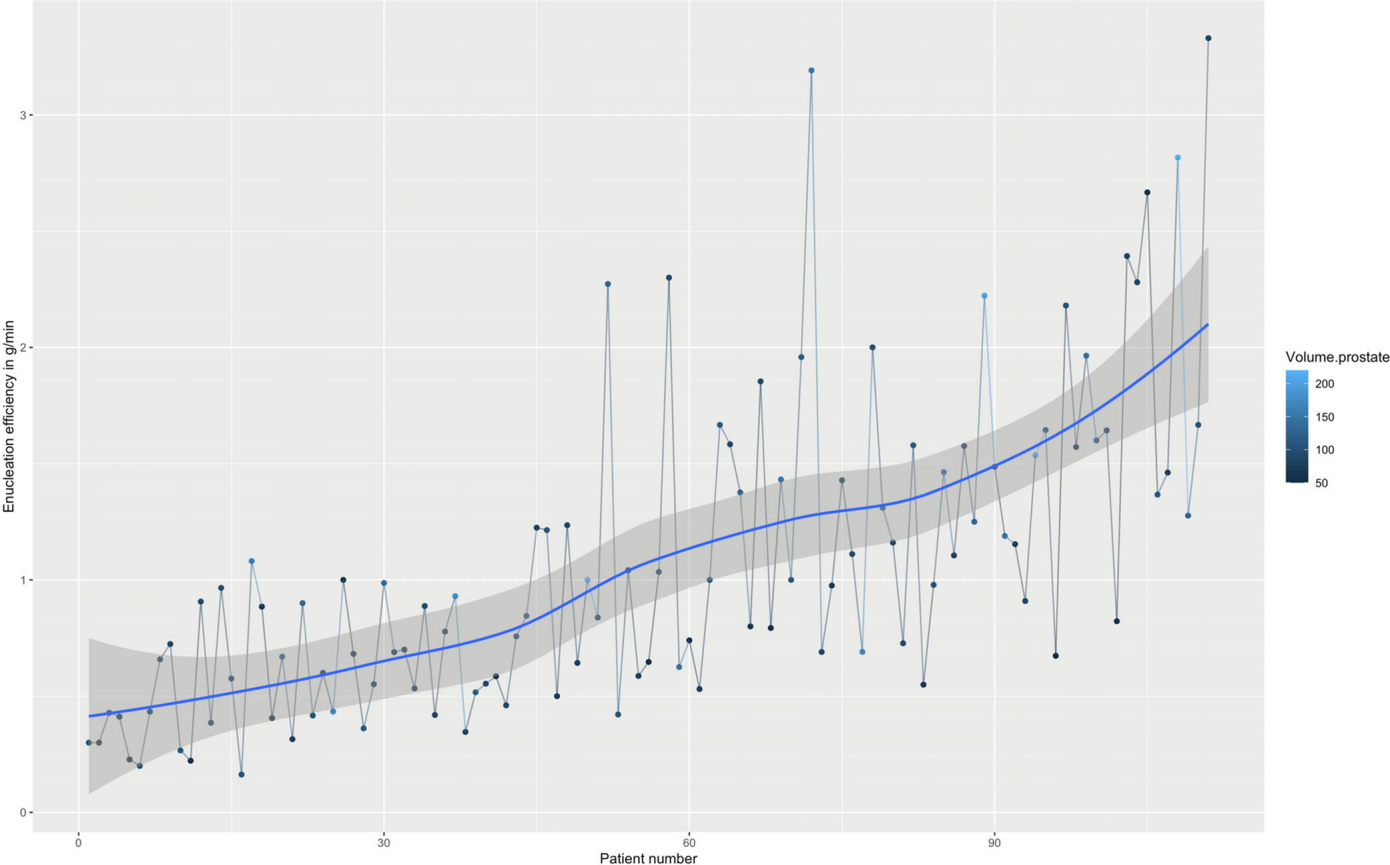
Evolution of enucleation efficiency over consecutive patients. The average (SD) enucleation efficiency was 1.1 g/min (SD = 0.7), and the plateau had not yet been reached after 111 patients.

Finally, operative and enucleation times decreased during the first procedures to reach a plateau after approximately 60 procedures (Figures 1 and 2). The average (SD) enucleation efficiency was 1.1 g/min (SD = 0.7), and the plateau had not yet been reached after 111 patients (Figure 3).

## 4 DISCUSSION

Our study aimed to evaluate the learning curve for THUFLEP performed by a single surgeon in a high‐volume academic centre. We assessed the learning curve using the pentafecta criteria and analysed perioperative outcomes over time. Our results suggest that the learning curve was overcome after approximately 40 cases, with operative and enucleation times stabilizing after 60 cases.

The learning curve for EEP has been extensively studied for HoLEP, with estimates ranging from 20 to 50 cases to achieve proficiency and approximately 60 cases to reach a plateau in operative efficiency.^8^, ^11^, ^13^ Enikeev et al. reported a learning curve of approximately 30–40 cases for ThuFLEP, with enucleation time and efficiency improving over time.^9^ Similarly, another recent study evaluating a single‐surgeon ThuFLEP experience in real‐world settings suggested that around 40–50 cases were required to achieve stable outcomes.^14^ Our findings align with these studies, reinforcing the notion that ThuFLEP has a learning curve comparable to HoLEP but may allow faster proficiency due to its shorter wavelength, enhanced tissue absorption and hence superior haemostatic properties.

Several factors were identified as influencing the learning curve. Our analysis showed that higher preoperative prostate volume and increased BMI were predictors of pentafecta failure. This finding is consistent with prior research evaluating HOLEP procedures and indicating that larger prostates require more intricate enucleation manoeuvres and longer morcellation times, potentially delaying proficiency acquisition.^15^, ^16^ Literature regarding the influence of prostate volume over the THUFLEP procedure remains mitigated. In their multicentre retrospective study including 2732 THUFLEP procedures, Castellani et al. evidenced that complications and postoperative urinary incontinence were not prostate volume dependent except for the blood transfusion rate, which was higher in the >80 ml prostate volume group.^17^ However, the authors evidenced that enucleation and morcellation times were greater in the >80 ml group, aligning with our study. In their study, Morozov et al. found similar results.^18^ The effect of BMI as a predictor of surgical difficulty due to technical challenges has been previously identified in very few studies. In 2020, Juvet et al. evidenced that a higher BMI was associated with an increased risk of open conversion during the HOLEP procedure.^19^ No data were found for THUFLEP procedures.

Finally, we did not find a study in the literature correlating directly pentafecta achievement with BMI or preoperative prostate volume.

In our study, the enucleation technique was not correlated with the pentafecta achievement and, the transition from a trilobe to an *en bloc* enucleation technique did not alter the learning curve. In their series, Saredi et al. demonstrated that *en bloc* enucleation could be safely performed by a surgeon experienced in the three‐lobe technique.^20^ The surgeon's performance remained stable after switching to the en bloc technique, suggesting that the experience acquired with the trilobe approach facilitated the adaptation to en bloc enucleation and prevented a second learning curve. Unfortunately, data are lacking in the literature to enable us to decide on the most optimal sequence for reaching a plateau.

From a clinical perspective, our findings support the feasibility of adopting ThUFLEP in high‐volume centres with structured training programmes. Given the comparable learning curve to HoLEP, urologists experienced in TURP may transition to ThUFLEP with an expected proficiency within 40–60 cases. Furthermore, our study highlights the importance of patient selection, as high BMI and mainly large prostate volume may necessitate additional training considerations. According to our personal experience, we should recommend starting the first patients with prostate volume comprised between 60 and 80 to reach the learning curve and to increase prostate volume step by step.

This study has some limitations. First, it is a single‐surgeon experience, which limits the generalizability of the findings. However, this design ensured homogeneity in surgical technique and perioperative management, allowing a more accurate assessment of an individual learning curve. Second, while we used pentafecta criteria to define learning curve achievement, other metrics such as self‐reported surgeon confidence and complication rates over time could further refine the analysis. Third, the study design was retrospective. Lastly, long‐term functional outcomes were not assessed beyond the 3‐month follow‐up period.

## 5 CONCLUSIONS

Our study demonstrates that the learning curve for ThuFLEP is comparable to other enucleation techniques, with proficiency achieved after approximately 40 cases and efficiency plateauing after 60 cases. Patient factors such as BMI and prostate volume influence the learning curve and should be considered when training surgeons in this technique. Further multicentre studies are needed to confirm these findings and establish standardized training protocols for optimizing the learning curve of ThuFLEP.

## AUTHOR CONTRIBUTIONS

**Sulaiman Goudaimy:** Writing—original draft. Louis Lenfant: Validation and methodology. **Christophe Vaessen:** Validation and methodology. **Jérôme Parra:** Validation and methodology. **Emmanuel Chartier‐Kastler:** Validation and methodology. **Aurélien Beaugerie:** Validation and methodology. **Pierre Mozer:** Validation and methodology. **Quentin Dubourg:** Validation and methodology. **Thomas Seisen:** Validation and methodology. **Morgan Roupret:** Conceptualization; validation and supervision. **Ugo Pinar:** Conceptualization, Validation; supervision; writing—review.

## CONFLICT OF INTEREST STATEMENT

None.
